# The complete chloroplast genome of *Camellia melliana* (Theaceae)

**DOI:** 10.1080/23802359.2026.2642523

**Published:** 2026-03-13

**Authors:** Mengyuan Xu, Quannian Li, Haiguang Gong, Shaoshan Luo, Jiuxiang Huang

**Affiliations:** College of Forestry and Landscape Architecture, South China Agricultural University, Guangzhou, China

**Keywords:** Chloroplast genome, phylogenetic analysis, plastome, theaceae

## Abstract

*Camellia melliana* Hand. -Mazz. is an endangered shrub species endemic to China, but it has not been sequenced and has never been included in molecular phylogenetic studies to date. In the present study, the complete chloroplast genome sequence of the species was assembled through the genome-skimming approach, and the phylogenetic position of the species within *Camellia* was investigated for the first time. Results showed that the chloroplast genome of the species is 156,984 bp in length, including a large single copy (LSC) region of 86,588 bp and a small single-copy (SSC) region of 18,268 bp, which were separated by a pair of inverted repeat (IR) regions of 26,064 bp. The genome encoded 112 unique genes, including 79 protein-coding genes, four ribosomal RNA genes and 29 transfer RNA genes. The overall GC content of the complete genome is 37.3%. Results from phylogenetic analysis recovered a highly supported sister relationship between *C. melliana* and *C. salicifolia*. This study provides a foundation for the phylogenetics, taxonomy and exploration of genetic diversity of *Camellia*.

## Introduction

*Camellia* L., comprising approximately 232 tree and shrub species, represents the largest genus within the tea family Theaceae (POWO 2025). Over 80% of its species diversity is concentrated in China (Chang and Ren 1998). According to Chang’s taxonomic system, Chinese *Camellia* species have been classified into four subgenera and 18 sections based on comprehensive morphological analyses of floral and fruit characteristics (Chang and Ren 1998). The genus holds significant economic and ornamental importance, with numerous species utilized for tea production, oil extraction, and horticultural purposes (Wu et al. 2022).

*Camellia melliana* Hand.-Mazz. 1922, an evergreen shrub endemic to Guangdong Province, China, belongs to section *Eriandria* Coh. St. according to Chang’s taxonomic system (Chang and Ren 1998). The species was listed as endangered in the Redlist of China’s Biodiversity (MEE 2023), while it represents an important component in evergreen broad-leaved forests in Guangdong Province (Zou et al. 2014). Although phylogenetic studies focusing on the large genus *Camellia* have been extensively conducted in recent years, *C. melliana* has remained unsequenced, and no DNA sequence data for this species have been reported to date. In the present study, we sequenced and analyzed the complete chloroplast genome of *C. melliana* and investigated its phylogenetic position within *Camellia* for the first time.

## Materials and methods

**Figure 1.**
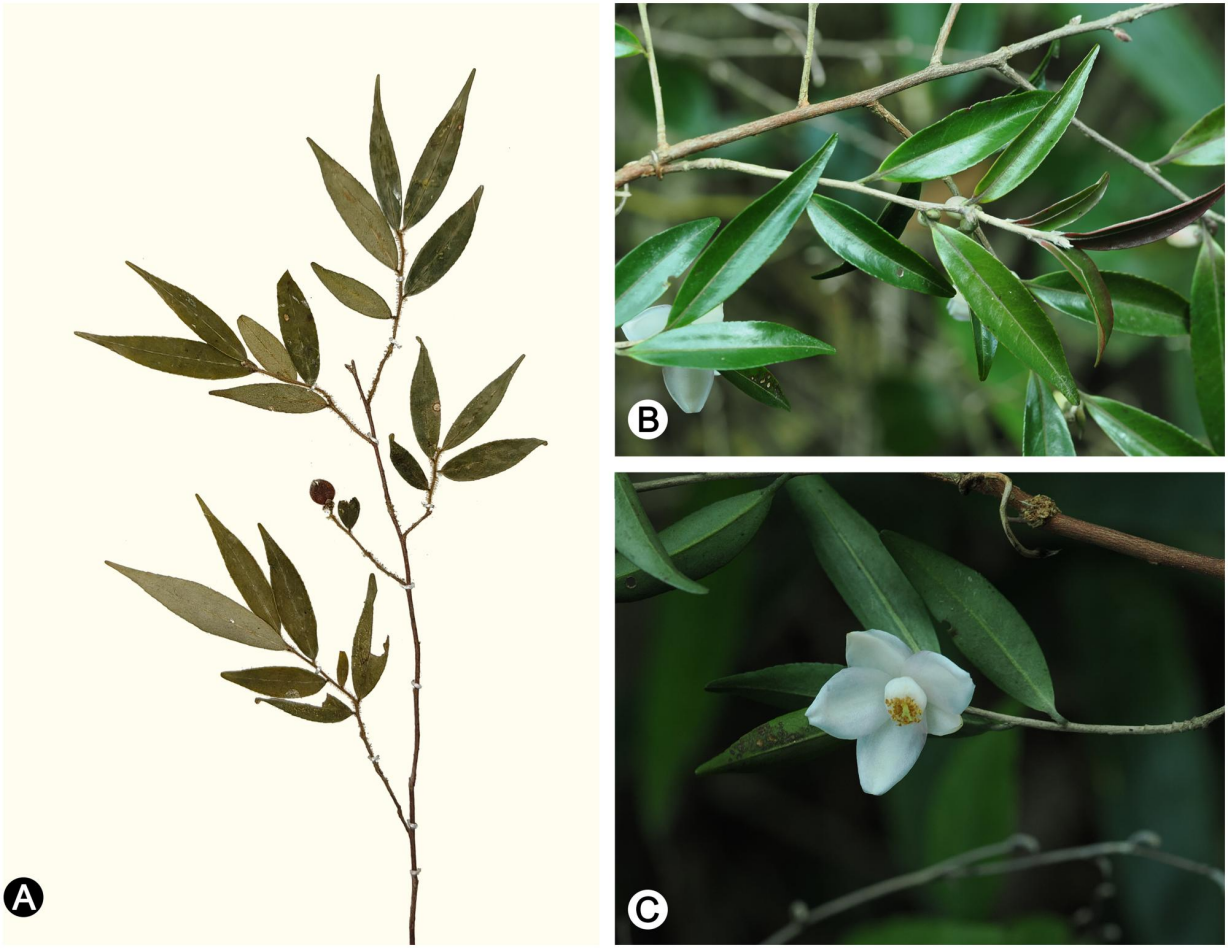
(A) The voucher specimen of *Camellia melliana*, collected from Chenhedong Provincial Nature Reserve in Guangzhou, Guangdong Province, China (prepared by Shaoshan Luo). Photographs of *Camellia melliana* taken by Yousheng Chen in Yangjiang City, Guangdong Province (B and C). Permission to use the photographs was obtained from Yousheng Chen. Diagnostic features of *Camellia melliana* include pubescent young branches, oblong-lanceolate leaves (3–5 cm long) with a bluntly apiculate apex, and white axillary flowers with 5–6 petals partially adnate to the stamens.

**Figure 2.**
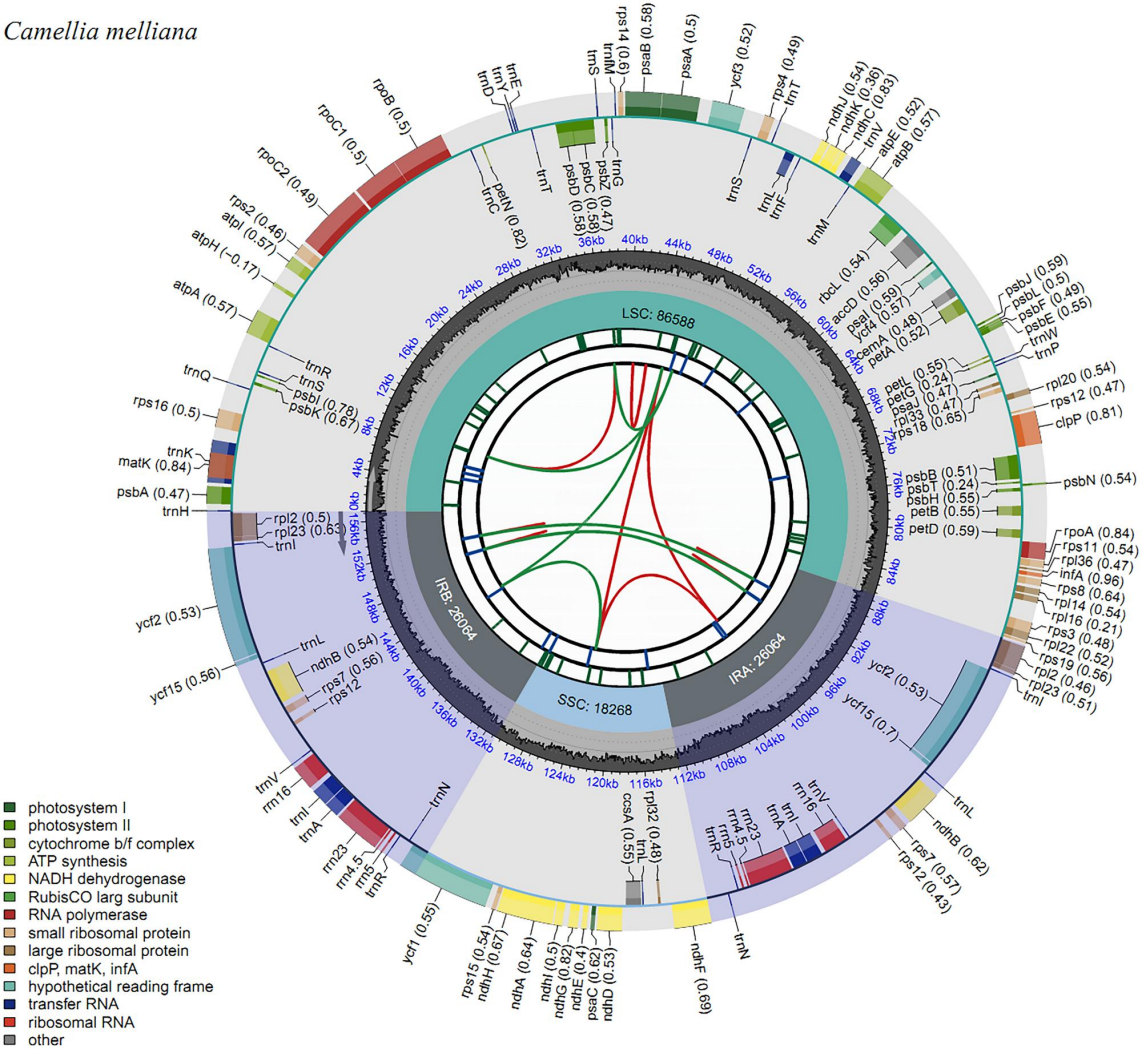
Schematic map of overall features of the chloroplast genome of *Camellia melliana*. The map contains six tracks in default. From the Center outward, the first track shows the dispersed repeats. The dispersed repeats consist of direct (D) and palindromic (P) repeats, connected with red and green arcs. The second track shows the long tandem repeats as short blue bars. The third track shows the short tandem repeats or microsatellite sequences as short bars with different colors. The small single-copy (SSC), inverted repeat (IRa and IRb), and large single-copy (LSC) regions are shown on the fourth track. The GC content along the genome is plotted on the fifth track. The genes are shown on the sixth track. The optional codon usage bias is displayed in the parenthesis after the gene name. Genes are color-coded by their functional classification which is shown in the bottom left corner. The transcription directions for the inner and outer genes are clockwise and anticlockwise, respectively.

Plant material of *Camellia melliana* was collected from the type locality of the species, viz. Chenhedong Provincial Nature Reserve in Guangzhou, Guangdong Province, China (N23°44′41.55″ E113°55′47.36″). The voucher specimen (Shaoshan Luo CHD20230812; Figure 1) was deposited in the Herbarium of South China Agricultural University (CANT; Index Herbariorum: https://sweetgum.nybg.org/science/ih/herbarium-details/?irn=126001; Curator: Prof. Yongbin Wu, email: ybwu@scau.edu.cn). Total genomic DNA was extracted from approximately 10 mg silica gel-dried leaf tissue using a modified CTAB protocol (Doyle and Doyle 1987). The chloroplast genome of *C. melliana* was sequenced through genome-skimming following the methodology of Xue et al. (2024). DNA was sheared to short fragments through ultrasonic treatment, and then the fragments approximately 500-bp in length were selected and used to construct short-insert library following the manufacturer’s protocol (NEBNext ® Ultra II ™DNA Library Prep Kit for Illumina®). Paired-end sequencing (2 × 150 bp) was performed on the Illumina HiSeq 2500 platform at Beijing Genomics Institute (BGI, Shenzhen, China), yielding approximately 3 GB of raw data. De novo assembly was conducted with GetOrganelle (Jin et al. 2020) using the chloroplast genome of *Camellia caudata* Wall. (OR333995) as a reference, which was chosen as a high-quality plastome from the same genus to facilitate accurate chloroplast read recruitment and assembly, given its completeness and annotation quality. Gene annotation was performed with the Plastid Genome Annotator (Qu et al. 2019). The complete chloroplast genome sequence has been deposited in NCBI GenBank (accession: PV345991; https://www.ncbi.nlm.nih.gov). A physical genome map was generated using CPGView (Liu et al. 2023; Figure 2).

To investigate the phylogenetic position of *Camellia melliana*, complete chloroplast genome sequences from 47 additional *Camellia* species were retrieved from the NCBI GenBank database (Supplementary material, Table 1). Three taxa from closely related genera, *Apterosperma* H.T. Chang, *Polyspora* Sweet and *Tutcheria* Dunn, were selected as outgroups based on the phylogenetic framework of Theaceae proposed by Yu et al. (2017). Eighty-three coding regions, including 79 protein-coding genes and four ribosomal RNA (rRNA) genes (Supplementary material, Table 2) in plastome were extracted and then aligned using the MAFFT algorithm (Katoh et al. 2019). The aligned gene matrices were concatenated and used to reconstruct the phylogenetic tree. Detailed information regarding these genes and their lengths are provided in Supplementary material, Table 2. The maximum likelihood (ML) approach implemented in RAxML version 8.1.24 (Stamatakis 2006) was used to infer the phylogenetic tree, employing the GTRGAMMA model with the default number of rate categories (C = 25). The model accounts for among-site rate heterogeneity and is widely used for robust plastome-scale ML phylogenetic inference. A rapid bootstrap (BS) analysis with 1000 pseudoreplicates was conducted to obtain support values for each phylogenetic node.

## Results

Structural analysis of the complete chloroplast genome of *Camellia melliana* revealed a typical quadripartite circular structure with 156,984 bp in length (Figure 2). The plastome exhibited an average read mapping depth of approximately 281× (Supplementary material, Figure 1). The final plastome assembly was gap-free (no ambiguous bases) and circularized. The genome comprises four distinct regions: a large single-copy (LSC) region of 86,588 bp, a small single-copy (SSC) region of 18,268 bp, and a pair of inverted repeat regions (IRa and IRb; 26,064 bp each). A total of 112 unique genes were annotated, including 79 protein-coding genes, four ribosomal RNA (rRNA) genes, and 29 transfer RNA (tRNA) genes. Several genes contained introns, including cis-splicing genes (e.g. *rpoC1*, *ycf3* and *clpP*) and the trans-splicing gene *rps12*, whose structures are shown in Supplementary material, Figure 2. Duplicated genes within the IR regions include seven protein-coding genes (*ndhB, rpl2, rpl23, rps12, rps7, ycf15, ycf2*), four rRNA genes (*rrn4.5, rrn5, rrn16, rrn23*), and seven tRNA genes (*trnA-UGC, trnL-CAA, trnI-CAU, trnI-GAU, trnN-GUU, trnR-ACG, trnV-GAC*). The overall GC content of the *C. melliana* chloroplast genome was calculated as 37.3%.

**Figure 3.**
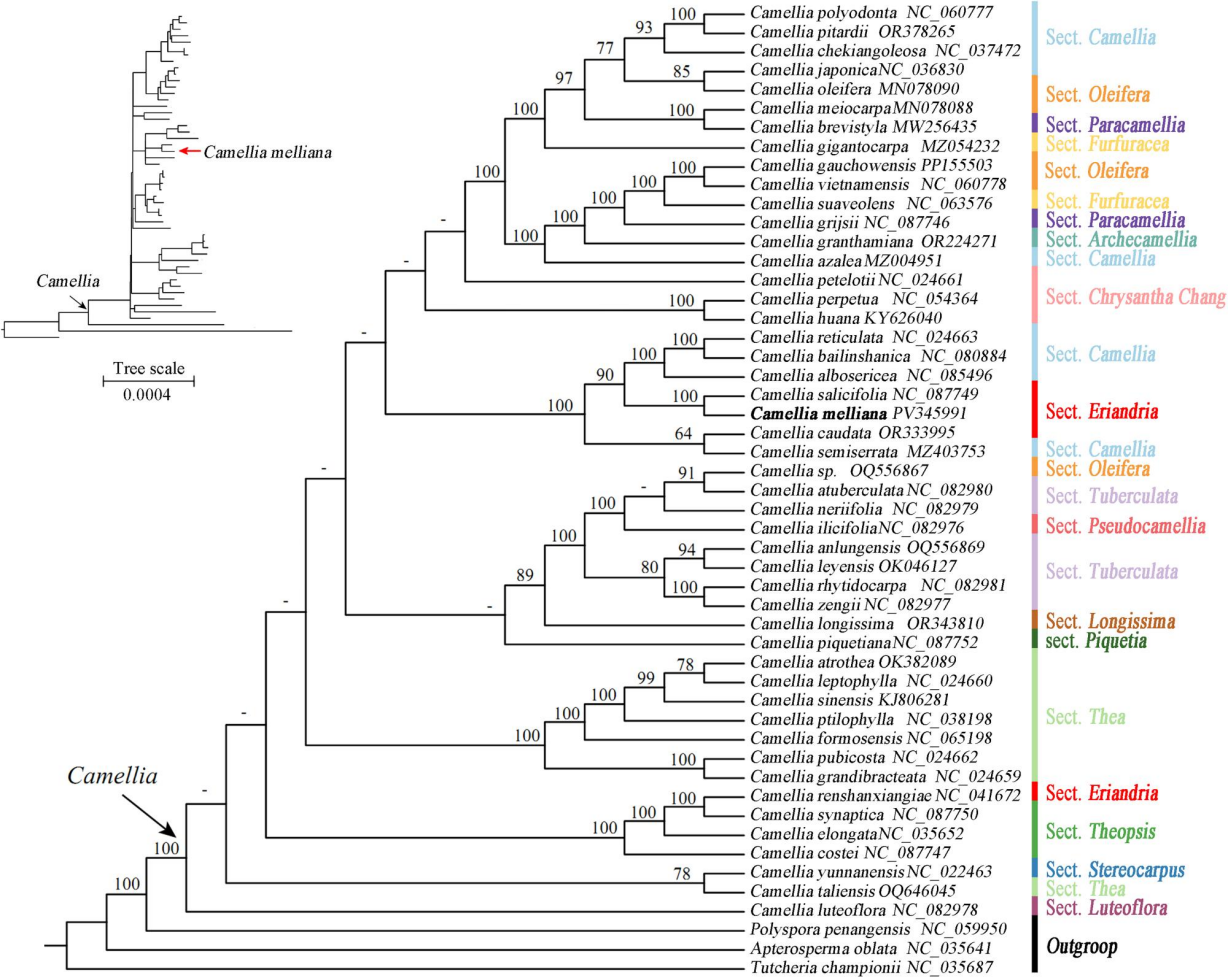
The maximum likelihood (ML) tree of sampled species of Theaceae based on analysis of 83 coding regions of chloroplast genomes. ML bootstrap percentages over 50% are given near the nodes, with dashes denoting a support inferior to 50%. Bold type marks species sequenced in the present study. Section assignments of *Camellia* species follow Chang and Ren (1998) and are indicated on the right of the cladogram. An inset phylogram with branch lengths proportional to substitutions per site (tree scale shown) is provided in the upper-left to complement the topology-focused main tree. Numbers following the species names represent GenBank accession numbers and the corresponding publications are as follows:*Camellia atrothea* (OK382089) (Wang et al. 2023); *Camellia azalea* (MZ004951) (Xu et al. 2023); *Camellia brevistyla* (MW256435) (Yin et al. 2021); *Camellia gigantocarpa* (MZ054232) (Xu et al. 2022); *Camellia ptilophylla* (NC_038198) (Li et al. 2018); *Camellia semiserrata* (MZ403753) (Dong et al. 2021); *Camellia anlungensis* (OQ556869), *Camellia leyensis* (OK046127) (Ran et al. 2024); *Camellia huana* (KY626040) (Wang et al. 2017); *Camellia leptophylla* (NC_024660), *Camellia petelotii* (NC_024661), *Camellia pubicosta* (NC_024662), *Camellia reticulata* (NC_024663), *Camellia synaptica* (NC_087750) and *Camellia taliensis* (OQ640645) (Huang et al. 2014); *Camellia sinensis* (KJ806281) and *Camellia yunnanensis* (NC_022463) (Yang et al. 2013); *Camellia granthamiana* (OR224271) (Chen et al. 2023). In addition, the following sequences were used as outgroups:*Polyspora penangensis* (NC_059950) (Choo et al. 2020), *Apterosperma oblata* (NC_035641) and *Tutcheria championii* (NC_035687) (Yu et al. 2017).

The phylogenetic analysis strongly supported the monophyly of the genus *Camellia* (BS = 100%) and revealed a well-supported sister relationship between the genus and *Polyspora* (BS = 100%; Figure 3). Notably, the species *C. melliana* formed a strongly supported sister clade (BS = 100%) with *C. salicifolia* Champ. ex Benth (Figure 3). *Camellia salicifolia* has been placed in sect. *Eriandria* in morphology-based classifications (Chang and Ren 1998) and the recovered relationship is therefore congruent with the sectional placement of *C. melliana*. This congruence also aligns with the general utility of plastome phylogenomics for resolving relationships among closely related *Camellia* taxa reported in previous studies. In addition, several major clades within *Camellia* were resolved with high statistical support, but the majority of backbone nodes in the genus exhibited weak phylogenetic signals and resolved with weak support. This topological ambiguity suggests that incorporating additional molecular markers, particularly nuclear genomic data, would be essential for elucidating evolutionary relationships among major lineages in this species-rich genus.

## Discussion and conclusion

Beyond phylogenetic inference, the newly generated plastome sequence also provides baseline genomic information for conservation-oriented assessments of threatened *Camellia* lineages. Given that several species in sect. *Eriandria* have been assessed as threatened in national red-list assessments, such genomic baseline resources may facilitate evidence-based conservation prioritization by improving taxonomic resolution and enabling downstream population genetic analyses. Accordingly, our data may serve as a reference for future reassessments and potential updates of protection priorities for threatened taxa.

In the present study, the endangered Chinese endemic species *Camellia melliana* was sequenced for the first time and its complete chloroplast genome sequence was provided. The phylogenetic position of the species within the large genus *Camellia* was also investigated here for the first time using molecular phylogenetic analysis, and a highly supported sister relationship between the species and *C. salicifolia* was recovered. This newly sequenced chloroplast genome provides valuable genomic data for advancing both conservation genetics and taxonomic studies of this species.

## Supplementary Material

Supplementary Tables.docx

Revised manuscript with highlights.docx

## Data Availability

The genome sequence data that support the findings of this study are openly available in GenBank of NCBI at https://www.ncbi.nlm.nih.gov under the accession no. PV345991. The associated BioProject, SRA, and Bio-Sample numbers are PRJNA1391648, SRR36546200, and SAMN54226502, respectively.
