# Supplementary material for: The complete chloroplast genome of Camellia melliana (Theaceae)
Source: Mitochondrial DNA B Resour. 2026 Mar 13;11(4):546–50. doi: 10.1080/23802359.2026.2642523 (PMC12990275; doi:10.1080/23802359.2026.2642523)
Supplement: Supplementary Tables.docx [file TMDN_A_2642523_SM5856.docx]

**Supplementary Table 1.** GenBank accession numbers of all the plastomes newly sequenced here as well as those obtained from NCBI ([www.ncbi.nlm.nih.gov](http://www.ncbi.nlm.nih.gov)).

| **Species** | **NCBI no.** |
| --- | --- |
| *Camellia albosericea* | NC_085496 |
| *Camellia anlungensis* | OQ556869 |
| *Camellia atrothea* | OK382089 |
| *Camellia atuberculata* | NC_082980 |
| *Camellia azalea* | MZ004951 |
| *Camellia bailinshanica* | NC_080884 |
| *Camellia brevistyla* | MW256435 |
| *Camellia caudata* | OR333995 |
| *Camellia chekiangoleosa* | NC_037472 |
| *Camellia costei* | NC_087747 |
| *Camellia elongata* | NC_035652 |
| *Camellia formosensis* | NC_065198 |
| *Camellia gauchowensis* | PP155503 |
| *Camellia gigantocarpa* | MZ054232 |
| *Camellia grandibracteata* | NC_024659 |
| *Camellia granthamiana* | OR224271 |
| *Camellia grijsii* | NC_087746 |
| *Camellia huana* | KY626040 |
| *Camellia ilicifolia* | NC_082976 |
| *Camellia japonica* | NC_036830 |
| *Camellia leptophylla* | NC_024660 |
| *Camellia leyensis* | OK046127 |
| *Camellia longissima* | OR343810 |
| *Camellia luteoflora* | NC_082978 |
| *Camellia meiocarpa* | MN078088 |
| *Camellia melliana* | PV345991 |
| *Camellia neriifolia* | NC_082979 |
| *Camellia oleifera* | MN078090 |
| *Camellia perpetua* | NC_054364 |
| *Camellia petelotii* | NC_024661 |
| *Camellia piquetiana* | NC_087752 |
| *Camellia pitardii* | OR378265 |
| *Camellia polyodonta* | NC_060777 |
| *Camellia ptilophylla* | NC_038198 |
| *Camellia pubicosta* | NC_024662 |
| *Camellia renshanxiangiae* | NC_041672 |
| *Camellia reticulata* | NC_024663 |
| *Camellia rhytidocarpa* | NC_082981 |
| *Camellia salicifolia* | NC_087749 |
| *Camellia semiserrata* | MZ403753 |
| *Camellia sinensis* | KJ806281 |
| *Camellia sp.* | OQ556867 |
| *Camellia suaveolens* | NC_063576 |
| *Camellia synaptica* | NC_087750 |
| *Camellia taliensis* | OQ646045 |
| *Camellia vietnamensis* | NC_060778 |
| *Camellia yunnanensis* | NC_022463 |
| *Camellia zengii* | NC_082977 |
| **Outgroups:** |  |
| *Apterosperma oblata* | NC_035641 |
| *Polyspora penangensis* | NC_059950 |
| *Tutcheria championii* | NC_035687 |

**Supplementary Table 2.** Sequence names and lengths for each of the coding regions used in reconstruction of phylogenetic tree.

| **Gene** | **Length** (bp) | **Gene** | **Length** (bp) |
| --- | --- | --- | --- |
| *accD* | 1,548 | *psbJ* | 123 |
| *atpA* | 1,524 | *psbK* | 186 |
| *atpB* | 1,497 | *psbL* | 117 |
| *atpE* | 402 | *psbM* | 105 |
| *atpF* | 567 | *psbN* | 132 |
| *atpH* | 246 | *psbT* | 117 |
| *atpI* | 744 | *rbcL* | 1,428 |
| *ccsA* | 966 | *rpl2* | 828 |
| *cemA* | 696 | *rpl14* | 369 |
| *clpP* | 645 | *rpl16* | 411 |
| *infA* | 234 | *rpl20* | 354 |
| *matK* | 1,500 | *rpl22* | 474 |
| *ndhA* | 1,092 | *rpl23* | 282 |
| *ndhB* | 1,533 | *rpl32* | 162 |
| *ndhC* | 363 | *rpl33* | 201 |
| *ndhD* | 1,515 | *rpl36* | 114 |
| *ndhE* | 306 | *rpoA* | 1,014 |
| *ndhF* | 2,256 | *rpoB* | 3,213 |
| *ndhG* | 531 | *rpoC1* | 2,061 |
| *ndhH* | 1,182 | *rpoC2* | 4,149 |
| *ndhI* | 504 | *rps2* | 711 |
| *ndhJ* | 477 | *rps3* | 657 |
| *ndhK* | 678 | *rps4* | 606 |
| *petA* | 963 | *rps7* | 468 |
| *petB* | 657 | *rps8* | 408 |
| *petD* | 534 | *rps11* | 417 |
| *petG* | 114 | *rps12* | 372 |
| *petL* | 96 | *rps14* | 303 |
| *petN* | 90 | *rps15* | 273 |
| *psaA* | 2,253 | *rps16* | 264 |
| *psaB* | 2,205 | *rps18* | 306 |
| *psaC* | 246 | *rps19* | 279 |
| *psaI* | 111 | *rrn4.5* | 103 |
| *psaJ* | 135 | *rrn5* | 121 |
| *psbA* | 1,062 | *rrn16* | 1,490 |
| *psbB* | 1,527 | *rrn23* | 2,809 |
| *psbC* | 1,422 | *ycf1* | 4,060 |
| *psbD* | 1,062 | *ycf2* | 6,915 |
| *psbE* | 252 | *ycf3* | 507 |
| *psbF* | 120 | *ycf4* | 555 |
| *psbH* | 222 | *ycf15* | 249 |
| *psbI* | 156 |  |  |
