# Supplementary material for: The complete chloroplast genome of Camellia melliana (Theaceae)
Source: Mitochondrial DNA B Resour. 2026 Mar 13;11(4):546–50. doi: 10.1080/23802359.2026.2642523 (PMC12990275; doi:10.1080/23802359.2026.2642523)
Supplement: Revised manuscript with highlights.docx [file TMDN_A_2642523_SM5854.docx]

The complete chloroplast genome of *Camellia melliana* (Theaceae)

Mengyuan Xu, Quannian Li, Haiguang Gong, Shaoshan Luo and Jiuxiang Huang*

College of forestry and landscape architecture, South China Agricultural University, Guangzhou, China

corresponding author: Jiuxiang Huang

email: jxhuang@scau.edu.cn

The complete chloroplast genome of *Camellia melliana* (Theaceae)

**Abstract:** *Camellia melliana* Hand. -Mazz. is an endangered shrub species endemic to China, but it has not been sequenced and has never been included in molecular phylogenetic studies to date. In the present study, the complete chloroplast genome sequence of the species was assembled through the genome-skimming approach, and the phylogenetic position of the species within *Camellia* was investigated for the first time. Results showed that the chloroplast genome of the species is 156,984 bp in length, including a large single copy (LSC) region of 86,588 bp and a small single-copy (SSC) region of 18,268 bp, which were separated by a pair of inverted repeat (IR) regions of 26,064 bp. The genome encoded 112 unique genes, including 79 protein-coding genes, four ribosomal RNA (rRNA) genes and 29 transfer RNA genes. The overall GC content of the complete genome is 37.3%. Results from phylogenetic analysis recovered a highly supported sister relationship between *C. melliana* and *C. salicifolia*. This study provides a foundation for the phylogenetics, taxonomy and exploration of genetic diversity of *Camellia*.

**Keywords:** Chloroplast genome; phylogenetic analysis; plastome; Theaceae.

# Introduction

*Camellia* L., comprising approximately 232 tree and shrub species, represents the largest genus within the tea family Theaceae (POWO 2025). Over 80% of its species diversity is concentrated in China (Chang et al., 1998). According to Chang's taxonomic system, Chinese *Camellia* species have been classified into four subgenera and 18 sections based on comprehensive morphological analyses of floral and fruit characteristics (Chang et al., 1998). The genus holds significant economic and ornamental importance, with numerous species utilized for tea production, oil extraction, and horticultural purposes (Wu et al., 2022).

*Camellia melliana* Hand.-Mazz. 1922, an evergreen shrub endemic to Guangdong Province, China, belongs to section *Eriandria* Coh. St. according to Chang’s taxonomic system (Chang et al., 1998). The species was listed as endangered in the Redlist of China’s Biodiversity (MEE 2023), while it represents an important component in evergreen broad-leaved forests in Guangdong Province (Zou et al., 2014). Although phylogenetic studies focusing on the large genus *Camellia* have been extensively conducted in recent years, *C. melliana* has remained unsequenced, and no DNA sequence data for this species have been reported to date. In the present study, we sequenced and analyzed the complete chloroplast genome of *C. melliana* and investigated its phylogenetic position within *Camellia* for the first time.

# Materials and methods

Plant material of *Camellia melliana* was collected from the type locality of the species, viz. Chenhedong Provincial Nature Reserve in Guangzhou, Guangdong Province, China (N23°44'41.55" E113°55'47.36"). The voucher specimen (Shaoshan Luo CHD20230812; Figure 1) was deposited in the Herbarium of South China Agricultural University (CANT; Index Herbariorum: <https://sweetgum.nybg.org/science/ih/herbarium-details/?irn=126001>; Curator: Prof. Yongbin Wu, email: ybwu@scau.edu.cn). Total genomic DNA was extracted from approximately 10 mg silica gel-dried leaf tissue using a modified CTAB protocol (Doyle et al., 1987). The chloroplast genome of *C. melliana* was sequenced through genome-skimming following the methodology of Xue et al. (2024). DNA was sheared to short fragments through ultrasonic treatment, and then the fragments approximately 500-bp in length were selected and used to construct short-insert library following the manufacturer’s protocol (NEBNext ® Ultra II ™DNA Library Prep Kit for Illumina®). Paired-end sequencing (2 × 150 bp) was performed on the Illumina HiSeq 2500 platform at Beijing Genomics Institute (BGI, Shenzhen, China), yielding approximately 3 GB of raw data. De novo assembly was conducted with GetOrganelle (Jin et al., 2020) using the chloroplast genome of *Camellia caudata* Wall. (OR333995) as a reference, which was chosen as a high-quality plastome from the same genus to facilitate accurate chloroplast read recruitment and assembly, given its completeness and annotation quality. Gene annotation was performed with the Plastid Genome Annotator (Qu et al., 2019). The complete chloroplast genome sequence has been deposited in NCBI GenBank (accession: PV345991; https://[www.ncbi.nlm.nih.gov](http://www.ncbi.nlm.nih.gov)). A physical genome map was generated using CPGView (Liu et al., 2023; Figure 2).

To investigate the phylogenetic position of *Camellia melliana*, complete chloroplast genome sequences from 47 additional *Camellia* species were retrieved from the NCBI GenBank database (Supplementary Table 1). Three taxa from closely related genera, *Apterosperma* H.T. Chang, *Polyspora* Sweet and *Tutcheria* Dunn, were selected as outgroups based on the phylogenetic framework of Theaceae proposed by Yu et al. (2017). Eighty-three coding regions, including 79 protein-coding genes and four ribosomal RNA (rRNA) genes (Supplementary Table 2) in plastome were extracted and then aligned using the MAFFT algorithm (Katoh et al., 2019). The aligned gene matrices were concatenated and used to reconstruct the phylogenetic tree. Detailed information regarding these genes and their lengths are provided in Supplementary Table 2. The maximum likelihood (ML) approach implemented in RAxML version 8.1.24 (Stamatakis 2006) was used to infer the phylogenetic tree, employing the GTRGAMMA model with the default number of rate categories (C = 25). **The model accounts for among-site rate heterogeneity and is widely used for robust plastome-scale ML phylogenetic inference.** A rapid bootstrap (BS) analysis with 1000 pseudoreplicates was conducted to obtain support values for each phylogenetic node.

# Results

Structural analysis of the complete chloroplast genome of *Camellia melliana* revealed a typical quadripartite circular structure with 156,984 bp in length (Figure 2). The plastome exhibited an average read mapping depth of approximately 281× (Supplementary Figure 1). The final plastome assembly was gap-free (no ambiguous bases) and circularized. The genome comprises four distinct regions: a large single-copy (LSC) region of 86,588 bp, a small single-copy (SSC) region of 18,268 bp, and a pair of inverted repeat regions (IRa and IRb; 26,064 bp each). A total of 112 unique genes were annotated, including 79 protein-coding genes, four ribosomal RNA genes, and 29 transfer RNA (tRNA) genes. Several genes contained introns, including cis-splicing genes (e.g. rpoC1, ycf3 and clpP) and the trans-splicing gene rps12, whose structures are shown in Supplementary Figure 2. Duplicated genes within the IR regions include seven protein-coding genes (*ndhB, rpl2, rpl23, rps12, rps7, ycf15, ycf2*), four rRNA genes (*rrn4.5, rrn5, rrn16, rrn23*), and seven tRNA genes (*trnA-UGC, trnL-CAA, trnI-CAU, trnI-GAU, trnN-GUU, trnR-ACG, trnV-GAC*). The overall GC content of the *C. melliana* chloroplast genome was calculated as 37.3%.

The phylogenetic analysis strongly supported the monophyly of the genus *Camellia* (BS = 100%) and revealed a well-supported sister relationship between the genus and Polyspora (BS = 100%; Figure 3). Notably, the species *C. melliana* formed a strongly supported sister clade (BS = 100%) with *C. salicifolia* Champ. ex Benth. (Figure 3). *Camellia salicifolia* has been placed in sect. *Eriandria* in morphology-based classifications (Chang et al., 1998) and the recovered relationship is therefore congruent with the sectional placement of *C. melliana*. This congruence also aligns with the general utility of plastome phylogenomics for resolving relationships among closely related *Camellia* taxa reported in previous studies. In addition, several major clades within *Camellia* were resolved with high statistical support, but the majority of backbone nodes in the genus exhibited weak phylogenetic signals and resolved with weak support. This topological ambiguity suggests that incorporating additional molecular markers, particularly nuclear genomic data, would be essential for elucidating evolutionary relationships among major lineages in this species-rich genus.

# Discussion and Conclusion

Beyond phylogenetic inference, the newly generated plastome sequence also provides baseline genomic information for conservation-oriented assessments of threatened *Camellia* lineages. Given that several species in sect. Eriandria have been assessed as threatened in national red-list assessments, such genomic baseline resources may facilitate evidence-based conservation prioritization by improving taxonomic resolution and enabling downstream population genetic analyses. Accordingly, our data may serve as a reference for future reassessments and potential updates of protection priorities for threatened taxa.

In the present study, the endangered Chinese endemic species *Camellia melliana* was sequenced for the first time and its complete chloroplast genome sequence was provided. The phylogenetic position of the species within the large genus *Camellia* was also investigated here for the first time using molecular phylogenetic analysis, and a highly supported sister relationship between the species and *C. salicifolia* was recovered. This newly sequenced chloroplast genome provides valuable genomic data for advancing both conservation genetics and taxonomic studies of this species.

# Acknowledgments

The authors thank Yousheng Chen for providing the photographs used in Figure 1.

# Author contributions statement

CRediT: M. Xu: Validation, Visualization, Writing – review & editing; Q. Li: Data curation, Formal analysis, Investigation, Methodology, Software, Writing – original draft; H. Gong: Conceptualization, Investigation, Methodology; S. Luo: Investigation, Validation; J. Huang: Conceptualization, Project administration, Supervision, Writing – review & editing.

# Ethical approval

This article does not contain any studies with human participants or ani-mals performed by any of the authors. In this experiment, we did not collect any human or animal samples.

# Disclosure statement

No potential conflict of interest was reported by the authors.

# Funding

This work was supported by the Forestry Science and Technology Innovation Project of Guangdong Province (2023KJCX003).

# Data availability statement

The genome sequence data that support the findings of this study are openly available in GenBank of NCBI at https://www.ncbi.nlm.nih.gov under the accession no. PV345991. The associated BioProject, SRA, and Bio-Sample numbers are PRJNA1391648, SRR36546200, and SAMN54226502, respectively.

# References

Chang, H.D., Ren, S.X. (1998). Theaceae. In: Wu, C.Y. (Ed.), Flora Reipublicae Popularis Sinicae. Science Press, Beijing, pp. 1–251.

Chen, S., Li, W., Li, W., Liu, Z., Shi, X., Zou, Y., Liao, W., Fan, Q. (2023). Population genetics of *Camellia granthamiana*, an endangered plant species with extremely small populations in China. Frontiers in Genetics 14, 1252148. https://doi.org/10.3389/fgene.2023.1252148.

China Ministry of Ecology and Environment (MEE). (2023). Red List of China’s Biodiversity – Higher Plants. Available online: https://www.mee.gov.cn/ (accessed 2023).

Choo, L.M., Niissalo, M.A., Leong, P.K., Khew, G.S. (2020). The complete plastome sequence of *Gordonia penangensis* Ridl. supports the transfer of Asian *Gordonia into* *Polyspora* (Theaceae). Phytotaxa 458(2), 159–166. https://doi.org/10.11646/phytotaxa.458.2.3.

Dong, L., Yin, X., Huang, B., Li, T., Huang, J.J., Wen, Q. (2021). The complete chloroplast genome of *Camellia semiserrata* Chi. (Theaceae), an excellent woody edible oil and landscaping species in South China. Mitochondrial DNA Part B 6(10), 3013–3015. https://doi.org/10.1080/23802359.2021.1976690.

Doyle, J.J., Doyle, J.L. (1987). A rapid DNA isolation procedure for small quantities of fresh leaf tissue. Phytochemical Bulletin 19, 11–15.

Huang, H., Shi, C., Liu, Y., Mao, S.Y., Gao, L.Z. (2014). Thirteen *Camellia chloroplast* genome sequences determined by high-throughput sequencing: genome structure and phylogenetic relationships. BMC Evolutionary Biology 14, 151. https://doi.org/10.1186/1471-2148-14-151.

Jin, J.J., Yu, W.B., Yang, J.B., Song, Y., DePamphilis, C.W., Yi, T.S., Li, D.Z. (2020). GetOrganelle: a fast and versatile toolkit for accurate de novo assembly of organelle genomes. Genome Biology 21, 241. https://doi.org/10.1186/s13059-020-02154-5.

Katoh, K., Rozewicki, J., Yamada, K.D. (2019). MAFFT online service: multiple sequence alignment, interactive sequence choice and visualization. Briefings in Bioinformatics 20(4), 1160–1166. https://doi.org/10.1093/bib/bbx108.

Li, W., Xing, F., Ng, W.L., Zhou, Y., Shi, X. (2018). The complete chloroplast genome sequence of *Camellia ptilophylla* (Theaceae): a natural caffeine-free tea plant endemic to China. Mitochondrial DNA Part B 3(1), 426–427. https://doi.org/10.1080/23802359.2018.1457996.

Liu, S.Y., Ni, Y., Li, J.L., Zhang, X.Y., Yang, H.Y., Chen, H.M., Liu, C. (2023). CPGView: a package for visualizing detailed chloroplast genome structures. Molecular Ecology Resources 23, 694–704. https://doi.org/10.1111/1755-0998.13729.

Plants of the World Online (POWO). (2025). Plants of the World Online. Royal Botanic Gardens, Kew. Available online: https://www.plantsoftheworldonline.org/ (accessed 15 May 2025).

Qu, X.J., Moore, M.J., Li, D.Z., Yi, T.S. (2019). PGA: a software package for rapid, accurate, and flexible batch annotation of plastomes. Plant Methods 15, 50. https://doi.org/10.1186/s13007-019-0435-7.

Ran, Z., Li, Z., Xiao, X., et al. (2024). Complete chloroplast genomes of 13 species of sect. *Tuberculata* Chang (*Camellia* L.): genomic features, comparative analysis, and phylogenetic relationships. BMC Genomics 25, 108. https://doi.org/10.1186/s12864-024-09982-w.

Stamatakis, A. (2006). RAxML-VI-HPC: maximum likelihood-based phylogenetic analyses with thousands of taxa and mixed models. Bioinformatics 22(21), 2688–2690. https://doi.org/10.1093/bioinformatics/btl446.

Wang, G., Luo, Y., Hou, N., et al. (2017). The complete chloroplast genomes of three rare and endangered camellias (*Camellia huana*, *C. liberofilamenta* and *C. luteoflora*) endemic to Southwest China. Conservation Genetics Resources 9, 583–585. https://doi.org/10.1007/s12686-017-0727-z.

Wang, Y., Huang, J., Xie, N., Zhang, D., Tong, W., Xia, E. (2023). The complete chloroplast genome sequence of *Camellia atrothea* (Ericales: Theaceae). Mitochondrial DNA Part B 8(4), 536–540. https://doi.org/10.1080/23802359.2023.2204972.

Wu, Q., Tong, W., Zhao, H., Ge, R., Li, R., Huang, J., Li, F., Wang, Y., Mallano, A.I., Deng, W. (2022). Comparative transcriptomic analysis unveils the deep phylogeny and secondary metabolite evolution of 116 *Camellia plants*. The Plant Journal 111, 406–421. https://doi.org/10.1111/tpj.15799.

Xu, Y., Liu, Y., Yu, Z., Jia, X. (2023). Complete chloroplast genome sequence of the long blooming cultivar *Camellia* ‘Xiari Qixin’: genome features, comparative and phylogenetic analysis. Genes 14(2), 460. https://doi.org/10.3390/genes14020460.

Xu, Y., Liu, Y., Jia, X. (2021). Complete chloroplast genome of a cultivated oil camellia species, *Camellia gigantocarpa*. Mitochondrial DNA Part B 7(1), 43–45. https://doi.org/10.1080/23802359.2021.2008836.

Xue, B., Huang, E., Zhao, G., Wei, R., Song, Z., Zhang, X., Yao, G. (2024). ‘Out of Africa’ origin of the pantropical staghorn fern genus *Platycerium* (Polypodiaceae) supported by plastid phylogenomics and biogeographical analysis. Annals of Botany 133, 697–709. https://doi.org/10.1093/aob/mcae003.

Yang, J.B., Yang, S.X., Li, H.T., Yang, J., Li, D.Z. (2013). Comparative chloroplast genomes of Camellia species. PLoS One 8(8), e73053. https://doi.org/10.1371/journal.pone.0073053.

Yin, X., Huang, B., Wang, B., Xu, L., Wen, Q. (2021). The complete chloroplast genome of *Camellia brevistyla* (Hayata) Coh. St. (Theaceae: Ericales) from China based on PacBio and Illumina data. Mitochondrial DNA Part B 6(8), 2246–2248. https://doi.org/10.1080/23802359.2021.1917320.

Yu, X.Q., Gao, L.M., Soltis, D.E., Soltis, P.S., Yang, J.B., Fang, L., Yang, S.X., Li, D.Z. (2017). Insights into the historical assembly of East Asian subtropical evergreen broadleaved forests revealed by the temporal history of the tea family. New Phytologist 215(3), 1235–1248. https://doi.org/10.1111/nph.14683.

Zou, W., Jiang, Y., Yin, G., Li, R., Yang, J., Wang, D. (2014). Study on characteristics of vegetation community in Shimen National Forest Park [in Chinese]. Ecological Science 33, 276–281.


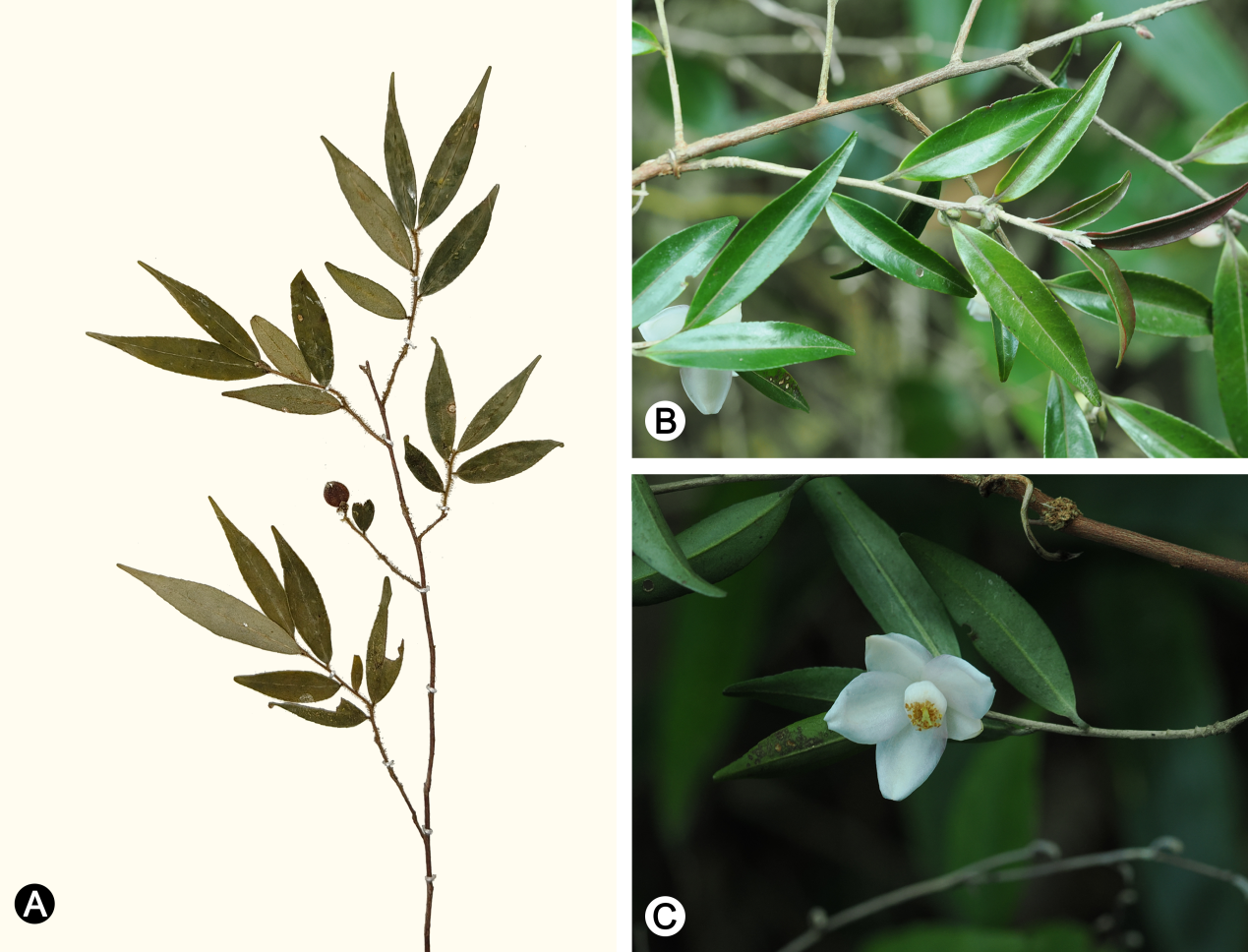


**Figure 1.** (A) The voucher specimen of *Camellia melliana*, collected from Chenhedong Provincial Nature Reserve in Guangzhou, Guangdong Province, China (prepared by Shaoshan Luo). Photographs of *Camellia melliana* taken by Yousheng Chen in Yangjiang City, Guangdong Province (B-C). Permission to use the photographs was obtained from Yousheng Chen. Diagnostic features of *Camellia melliana* include pubescent young branches, oblong-lanceolate leaves (3-5 cm long) with a bluntly apiculate apex, and white axillary flowers with 5-6 petals partially adnate to the stamens.


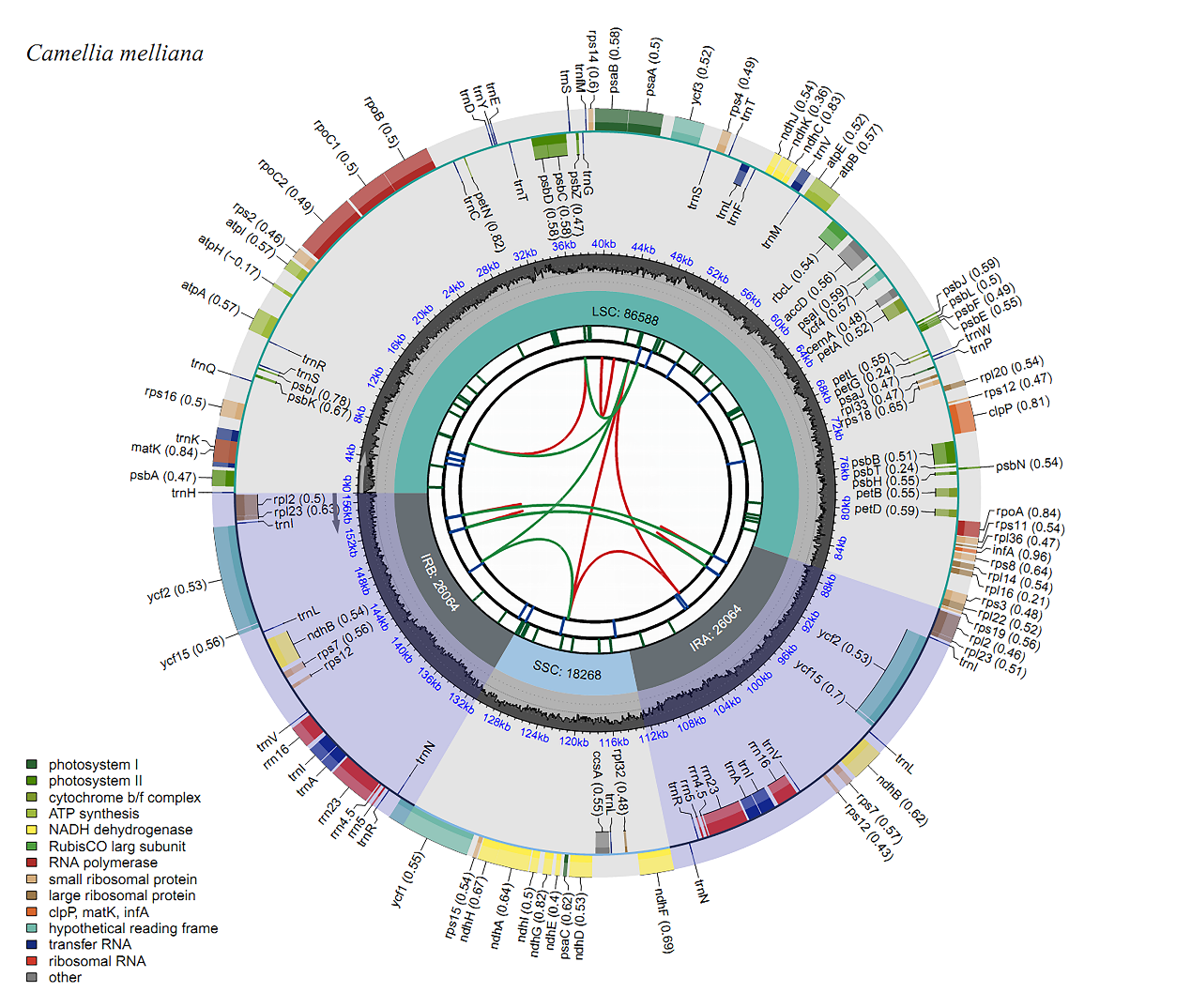


**Figure 2.** Schematic map of overall features of the chloroplast genome of *Camellia melliana*. The map contains six tracks in default. From the Centre outward, the first track shows the dispersed repeats. The dispersed repeats consist of direct (D) and palindromic (P) repeats, connected with red and green arcs. The second track shows the long tandem repeats as short blue bars. The third track shows the short tandem repeats or microsatellite sequences as short bars with different colors. The small single-copy (SSC), inverted repeat (IRa and IRb), and large single-copy (LSC) regions are shown on the fourth track. The GC content along the genome is plotted on the fifth track. The genes are shown on the sixth track. The optional codon usage bias is displayed in the parenthesis after the gene name. Genes are color-coded by their functional classification which is shown in the bottom left corner. The transcription directions for the inner and outer genes are clockwise and anticlockwise, respectively.


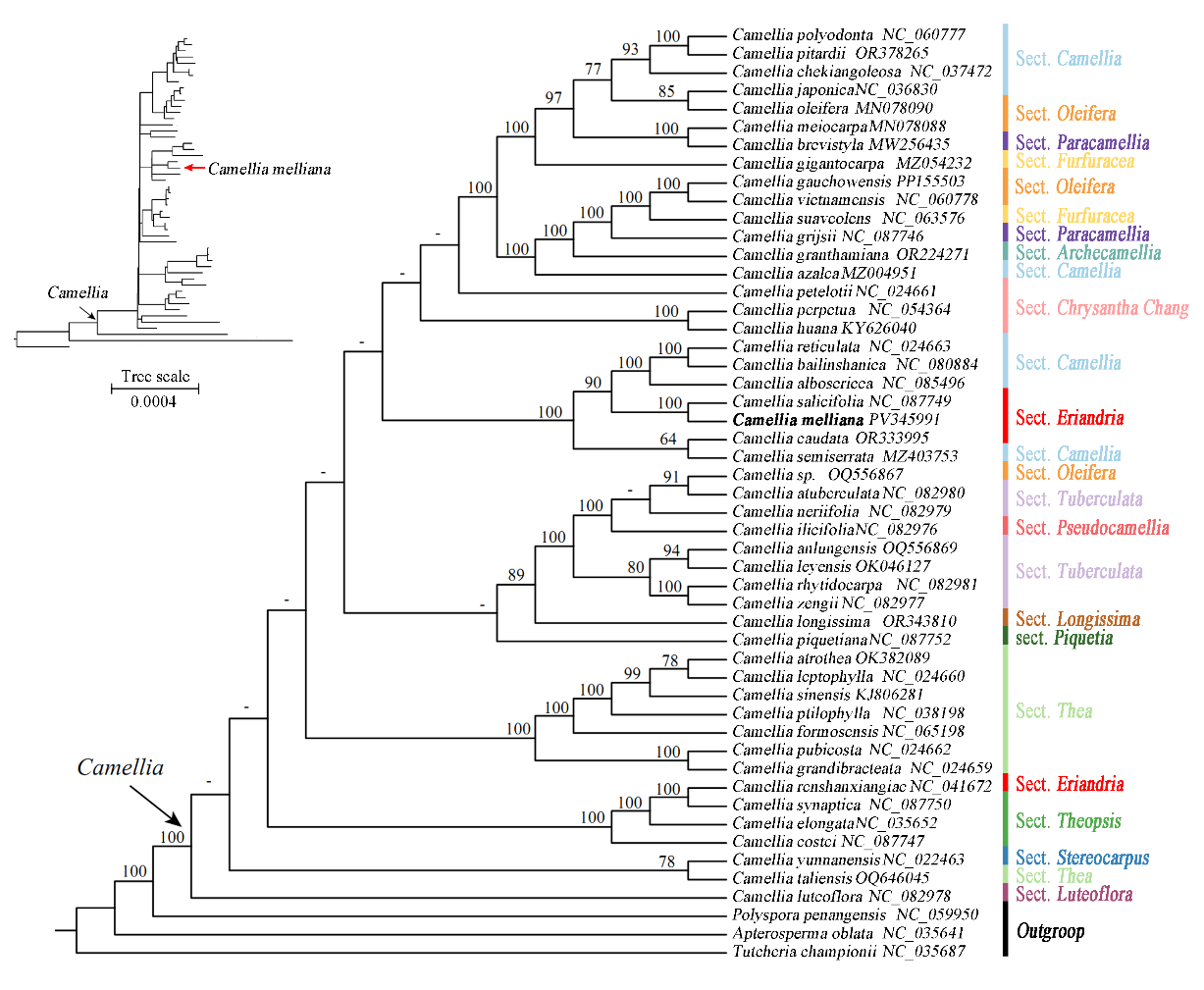


**Figure 3.** The maximum likelihood (ML) tree of sampled species of Theaceae based on analysis of 83 coding regions of chloroplast genomes. ML bootstrap percentages over 50% are given near the nodes, with dashes denoting a support inferior to 50%. Bold type marks species sequenced in the present study. Section assignments of *Camellia* species follow Chang et al. (1998) and are indicated on the right of the cladogram. An inset phylogram with branch lengths proportional to substitutions per site (tree scale shown) is provided in the upper-left to complement the topology-focused main tree. Numbers following the species names represent GenBank accession numbers and the corresponding publications are as follows:*Camellia atrothea* (OK382089) (Wang et al., 2023); *Camellia azalea* (MZ004951) (Xu et al., 2023); *Camellia brevistyla* (MW256435) (Yin et al., 2021); *Camellia gigantocarpa* (MZ054232) (Xu et al., 2021); *Camellia ptilophylla* (NC_038198) (Li et al., 2018); *Camellia semiserrata* (MZ403753) (Dong et al., 2021); *Camellia anlungensis* (OQ556869), *Camellia* leyensis (OK046127) (Ran et al., 2024); *Camellia huana* (KY626040) (Wang et al., 2017); *Camellia leptophylla* (NC_024660), *Camellia* *petelotii* (NC_024661), *Camellia* *pubicosta* (NC_024662), *Camellia* *reticulata* (NC_024663), *Camellia* *synaptica* (NC_087750) and *Camellia taliensis* (OQ640645) (Huang et al., 2014); *Camellia sinensis* (KJ806281) and *Camellia yunnanensis* (NC_022463) (Yang et al., 2013); *Camellia granthamiana* (OR224271) (Chen et al., 2023). In addition, the following sequences were used as outgroups:*Polyspora penangensis* (NC_059950) (Choo et al., 2020), *Apterosperma oblata* (NC_035641) and *Tutcheria championii* (NC_035687) (Yu et al., 2017).

**
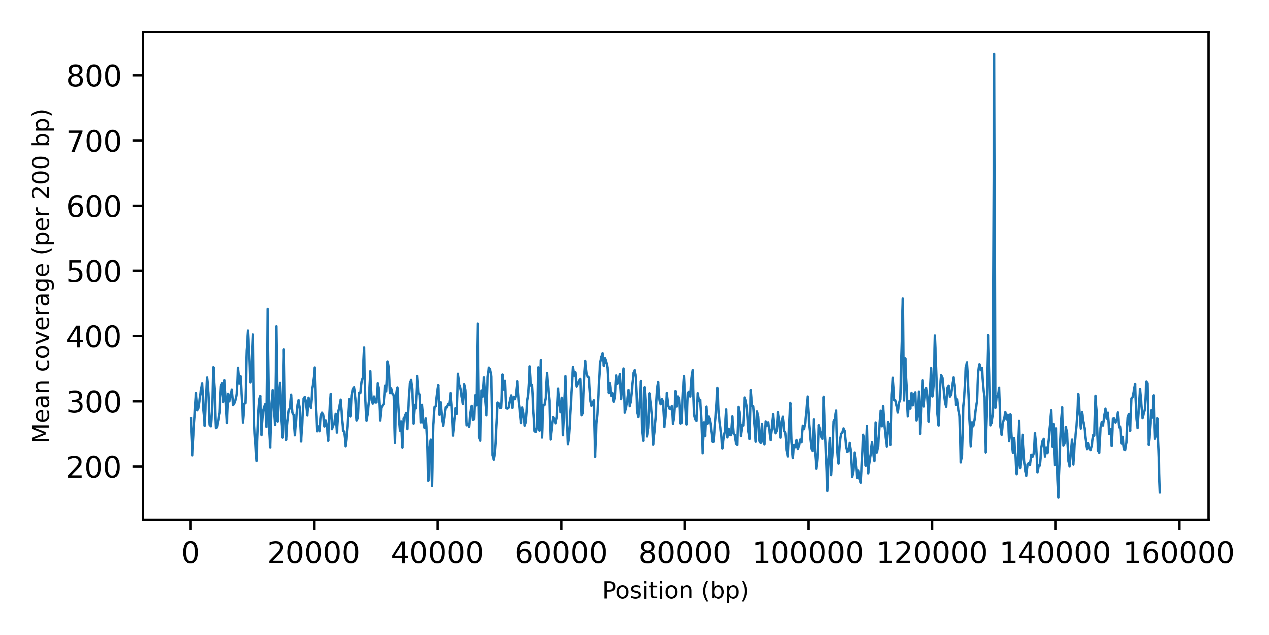
**

**Supplementary Figure 1.** Read coverage across the complete chloroplast genome of Camellia melliana. The minimum, maximum, and average sequencing depths were 99×, 6324×, and 281×, respectively.

**
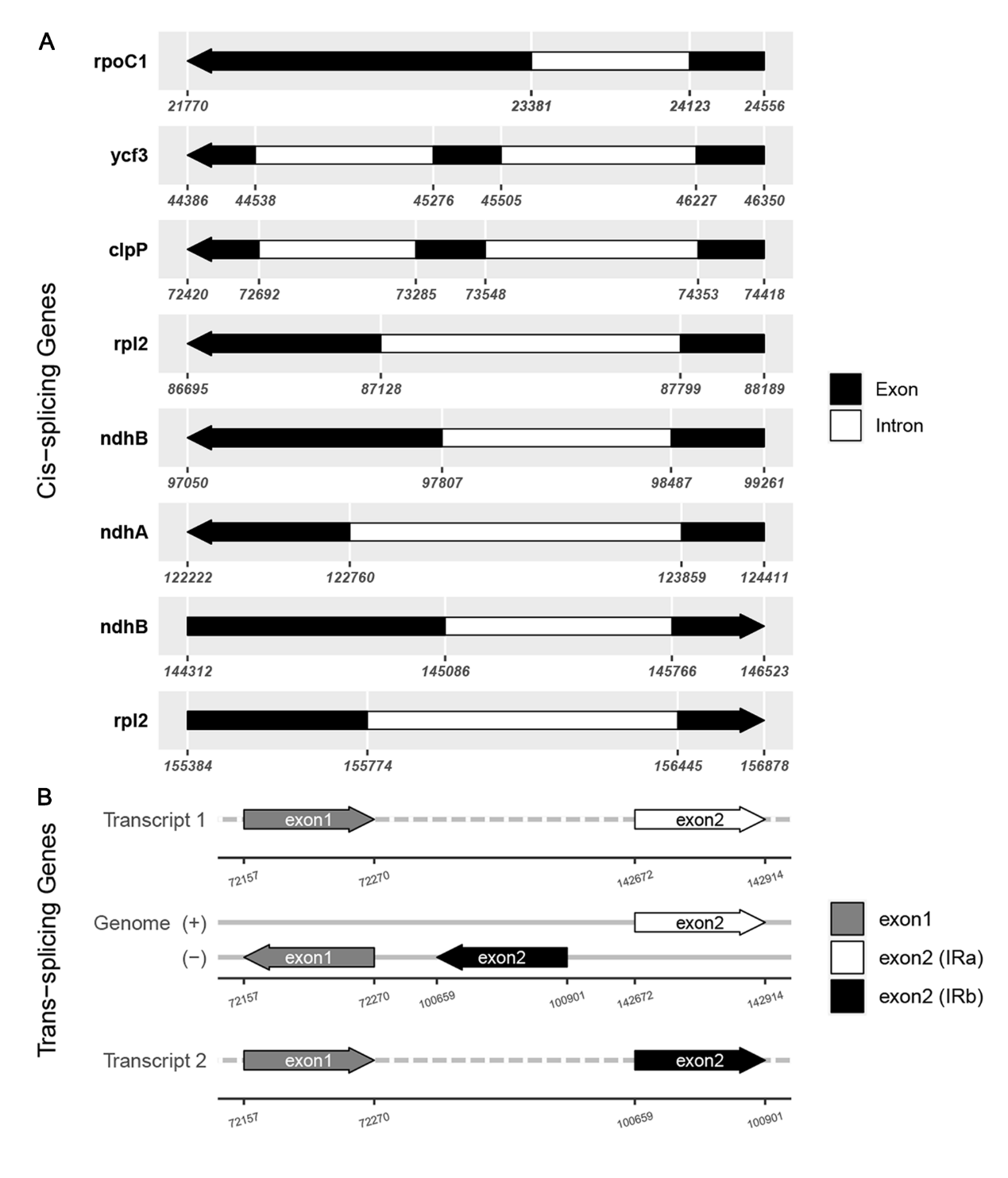
**

**Supplementary Figure 2.** Gene structures of cis-splicing (A) genes and the trans-splicing gene *rps12* (B) in the chloroplast genome of Camellia melliana.
